# Ultrasound-Assisted Extraction of Antioxidant Compounds from “Cẩm” Purple Rice Bran for Modulation of Starch Digestion

**DOI:** 10.1155/2023/1086185

**Published:** 2023-11-16

**Authors:** Le Thi Kim Loan, Nguyen Minh Thuy, Ngo Van Tai

**Affiliations:** ^1^Faculty of Agriculture and Food Technology, Tien Giang University, Tien Giang Province, Vietnam; ^2^Institute of Food and Biotechnology, Can Tho University, Can Tho City, Vietnam; ^3^School of Food Industry, King Mongkut's Institute of Technology Ladkrabang, Bangkok 10520, Thailand

## Abstract

Purple rice, locally known as “Cẩm” rice, is cultivated in the southern region of Vietnam. The bran of “Cẩm” rice is often disregarded and underutilized; nevertheless, it harbours substantial nutritive value, particularly in terms of antioxidant compounds. Additionally, sonication, an emerging and “green” technological approach, has been employed to augment the extraction efficiency of these antioxidants. This research is aimed at optimizing and maximizing the antioxidant recovery capacity including phenolic and total flavonoid compounds, along with their antioxidant activities, through the assistance of ultrasound waves. The effect of the extract on the starch digestion process was also investigated. The study employed the Box-Behnken experimental design, encompassing three variables: extraction time (20-40 minutes), temperature (60-80°C), and solvent-to-material ratio (8 : 1 to 12 : 1). Analysis was conducted on total phenolic compounds, total flavonoid content, and antioxidant activities. Results demonstrated that the peak yield of antioxidant compounds and their corresponding activities were attained at an extraction duration of 29.38 minutes, a temperature of 69°C, and a solvent-to-material ratio of 9.92. Under these optimal conditions, the yields were as follows: total phenolic compounds at 60.821 mg GAE/g, total flavonoid compounds at 3.2696 mg QE/g, percentage inhibition of DPPH at 74.778%, and FRAP value at 54.112 *μ*mol Fe (II)/g. The established models were validated and exhibited a strong alignment between predicted and actual values, with disparities of less than 3% under optimal conditions. Furthermore, the extract was codigested with cooked corn starch, revealing a dose-dependent effect on starch digestibility. The sluggishness of digestion rate was observed when 20 mg of the extract was supplemented to 200 mg of cooked corn starch. This suggests that rice bran extract holds promise as an effective ingredient for mitigating starch digestion, particularly beneficial for individuals dealing with diabetes.

## 1. Introduction

“Cẩm” rice, originating from Cai Lay district, Tien Giang province, is a type of rice with a higher content of nutrients than other types of white rice [1, 2]. Compared with white rice, brown rice contains a variety of minerals such as iron, zinc, phosphorus, vitamin B_1,_ and soluble fibre. Especially in the silk skin of “Cẩm,” rice has a high anthocyanin content [3]. However, it was considered as the waste after milling process [4]. The presence of significant amounts of bioactive substances, including dietary fibre, tocopherols, tocotrienols, oryzanols, phytic acid, and phenolic compounds, is usually thought to be responsible for these qualities [5–7]. Bioactive compounds were recently attracted by the consumer, especially after the COVID-19 pandemic, which could provide various health benefits [8, 9]. Due to its positive benefits on diabetes, the immune system, cancer, and heart disease, rice bran, which is separated from entire rice grains, has attracted a lot of interest [10, 11]. Recent study on the rice bran showed many potential to recover the antioxidant and nutrients [12, 13].

In addition, various methods were employed to enhance yield extraction. The conventional method is the most popular method for rice bran antioxidant because it is simple, easy to operate, and gives higher extractability [12]. However, this method requires a long extraction time and low capacity. Ultrasound-assisted extraction was found as one of the promising methods for producing high-quality extract with a high level of maintenance antioxidant compound [14, 15]. The majority of ultrasonic wave effects are caused by cavitation phenomena (physical effect) and/or generation of free radicals (chemical effect) [16, 17]. Recently, the ultrasound-assisted extraction was applied in various rich-polyphenols sources such as durian leaf waste [18], habanero pepper leaves (*Capsicum chinense*) [19], and *Ficaria kochii* [20]. It has shown that the ultrasound wave could promote the extraction yield of antioxidant compounds.

Besides, antioxidant compounds were found to enhance the immune function and antidiabetic and can be used as a therapeutic safety agent. Moreover, Ngo et al. [21] recently reported that antioxidant compounds could reduce the glycemic index of various ingredients/products. Corn is an important food crop, and corn starch accounts for about 46.3 of yellow corn flour, which is also widely applied in the food industry [22]. However, the high proportion of rapid digestible starch in corn starch is suggested to cause the rapid increase in blood sugar level after meal [23]. There are no earlier reports on the use of ultrasound-assisted extraction for the obtention of phytochemicals with antioxidant capacity from “Cẩm” rice bran from Vietnam. Moreover, there were limited researches about the effect of pigment extract from rice bran on corn starch digestibility. Therefore, this study is aimed at investigating the optimal conditions for the extraction of the antioxidant compound from local purple rice bran in Vietnam and then applied to study its behavior when codigestion with corn starch. This study could promote the usage of rice bran in the future, besides helping to establish the method or product for the diabetic person.

## 2. Materials and Methods

### 2.1. Materials

“Cẩm Cai Lậy” rice, known as purple rice, was purchased from the local market in Tien Giang province (Vietnam). Then, bran was collected after grinding mill at the local company. Purple rice bran was defatted by hexane and milled to pass the 100 mesh sieve to uniform particle size for further extraction [24].

### 2.2. Chemical and Reagents

Folin–Ciocalteu's phenols reagent, sodium carbonate, 2,3,5-triphenyltetrazolium chloride (TPTZ), 2,2-diphenyl-1-picrylhydrazyl (DPPH), hydrochloric acid, iron chloride, sodium acetate, pepsin from porcine gastric mucosa (P7000, ≥250 U/mg), bile salt (B8756), and pancreatin from porcine pancreas (P7545, 8 × USP) were purchased from Sigma-Aldrich (St Louis, MO, USA). Other reagents were purchased from Merck. All reagents in this study were of analytical grade, and Milli-Q water was used for the analyses.

### 2.3. Extraction Design

An ultrasound bath (M2800H-J, 110 W, Japan) was used for operating the extraction process. Based on the preliminary study, the optimization of the extraction process was carried out. Three selected parameters included extraction time (*X*_1_, 20-40 min), extraction temperature (*X*_2_, 60-80°C), and ratio of solvent to material (*X*_3_, 8-12 ml/mg). The optimization study was performed employing the Box-Behnken design (BBD, 3-factor, 3-level) based on 3^3^ factorial designs as shown in Table 1. BBD was applied for experimental design with 6 center points, and the total runs were 18. Briefly, the bran was weighed in a centrifuge tube based on the experimental design and added to the extraction solvent. The solvent used for extraction is 60% ethanol (food grade) [25]. After finishing the extraction process, the tube was centrifuged at 10000 rpm and filtered. The solvent was evaporated and dissolved with 80% methanol before using for further analysis.

### 2.4. Determination of Total Phenolic Content

Total phenolic content (TPC) was analysed using the Folin–Ciocalteu assay, which followed the method described by Wanyo et al. [26]. Briefly, 300 *μ*L of rice bran extract was reacted with 2.25 mL of 10% Folin–Ciocalteu reagent and allowed to stand at room temperature for 5 min; 2.25 mL of sodium carbonate (60 g/L) solution was added to the mixture. After 90 min at room temperature, the absorbance of the reaction was read at a wavelength of 725 nm. The standard curve of gallic acid was used for the calculation of the TPC of the extract.

### 2.5. Determination of Total Flavonoid Content

Total flavonoid content (TFC) was determined following the method of Wang et al. [27]. Briefly, 0.5 mL of the extract was mixed with 2.25 mL of distilled water in a test tube followed by the addition of 0.15 mL of 5% NaNO_2_ solution. After 6 min, 0.3 mL of a 10% AlCl_3_·6H_2_O solution was added and allowed to stand for another 5 min before 1.0 mL of 1 M NaOH was added. The mixture was mixed by vortex mixer. The extract reacted with aluminium chloride to form the yellow solution. The absorbance of the reaction was recorded at 415 nm. TFC was calculated using the standard curve of quercetin, expressed as milligram quercetin equivalent per gram (mgQE/g).

### 2.6. Determination of Antioxidant Activity

Ferric reducing antioxidant power (FRAP) assay and DPPH assay were used for the determination of the antioxidant activity of purple rice bran extract. For the FRAP assay, the method followed the description of Tabaraki and Nateghi [28]. Briefly, 50 *μ*L of extract was used for reaction with 1.5 mL of the FRAP reagent (sodium acetate buffer (300 mM, pH 3.6), 10 mM TPTZ solution in 40 mM HCl, and 20 mM FeCl_3_ solution in proportions of 10 : 1 : 1 (*v*/*v*), respectively). The UV-Vis spectrophotometer (Shimadzu UV18000, Japan) was used to record the value of the mixture's absorbance at 593 nm after incubation for 4 minutes. Three replicates were applied, and the standard curve of FeSO_4_ solution was developed to calculate the FRAP value of treatment. The results were expressed as *μ*mol Fe(II)/g dry weight of rice bran.

For the DPPH assay, 20 *μ*L of extract reacted with 180 *μ*L DPPH solution (0.1 mM in methanol 80%). The mixture was then observed value of absorbance at the wavelength of 517 nm. The percentage of inhibition was calculated.

### 2.7. Codigestion Corn Starch with Rice Bran Extract

The extract was processed under optimal conditions, which was used for the codigestion process with corn starch to understand the antidiabetic activities. After extraction, the extract was evaporated using an evaporator system (Buchi R-300, Japan).

The digestion process of corn starch was carried out with the addition of rice bran extract and adapted with slight modification from the method of Dhital et al. [29]. Briefly, 200 mg corn starch was accurately weighed, and 2 mL distilled water was added to cook at boiling water for 30 minutes. Different levels of purple rice bran extract were added for the codigestion experiment, including 0 mg (0%), 5 mg (2.5%), 10 mg (5%), 15 mg (7.5%), and 20 mg (10%). Then, artificial saliva, pepsin, pancreatin, and amyloglucosidase as *in vitro* stimulated digestion process were then added. The 0.1 mL digested solution was taken out at 0, 20, 120, 150, and 180 mins of the small intestinal phase. The released glucose was analysed by GOPOD kit from Megazyme to calculate the percentage of starch hydrolysis.

### 2.8. Data Analysis

All the analysis was carried out in triplicates, and the experimental results were expressed as means ± standard deviation. The extraction optimization results were analysed using the Statgraphics XV.I program. Experimental data were fitted to the following second-order polynomial model, and regression coefficients were obtained. The output of the optimization stage is the recommendation of several new formulas that are optimal based on the program. The significance of the mathematical model was verified using branched statistical analysis of variance inference (ANOVA), which was used to identify the linear models, quadratic models, and interaction regression coefficients for each response. The significances of all terms in the polynomial were analysed statistically by computing the *F* value at a probability (*P* value) of 0.05. The optimal point also was selected by the application with the aim to maximize all responses.

## 3. Results and Discussion

### 3.1. Model Fitting of the Extraction Process from Purple Rice Bran

#### 3.1.1. Multiple Linear Regressions and the Model's Adequacy

In some recent studies, the vital factors that most affected the effectiveness of ultrasound-assisted extraction were time, temperature, power, a ratio of material and solvent, and pH [30]. In this study, the food-grade ethanol solvent was used for extraction; besides that, the ultrasound bath also had constant operation power as a fixed factor. It can be seen from Table 2 that all factors affected the yield of antioxidant compounds. The total phenolic compound, total flavonoid content, FRAP value, and percentage of DPPH inhibition ranged from 45.111 to 61.808 mgGAE/g, 2.168 to 3.351 mgQE/g, 37.592 to 54.453 *μ*molFe(II)/g, and 60.549 to 75.137%, respectively.

In addition, Table 3 shows an analysis of variance for the determination of optimization model fit and regression coefficient values of the experimental variables. It shows that all the responses analysed provided significant models with a coefficient of determination (*R*^2^) above 90% indicating that the relationship between independence process variables and response is significant. Good reliability and high degrees of precision of the conducted experiments were implemented by the low value of the coefficient of variance. Besides, significance was found when the *P* value of models was lower than 0.05. In addition, the lack-of-fit test was shown not significantly different (*P* value > 0.05), which could confirm that the established models could be enough for predicting the extraction yield of antioxidant compounds and antioxidant activities of purple rice bran extract. The significant difference in the *P* value of model denotes the determination of the deviation in the output or response that can be pointed out by regression model equations [25, 31].

All the experimental data were fitted to the quadratic second-order polynomial mathematical model, and the final equations developed are given below:

(1) $$\begin{array}{c} TPC=-434.31+2.9267X_{1}+6.5341X_{2}+45.752X_{3}-0.026X_{1}X_{2}+0.0317X_{1}X_{3}-0.05X_{2}X_{3}-0.0256X_{1}^{2}-0.038X_{2}^{2}-2.183X_{3}^{2},\\ TFC=-36.468+0.2947X_{1}+0.582X_{2}+3.0372X_{3}-0.0016X_{1}X_{2}+0.0019X_{1}X_{3}-0.0005X_{2}X_{3}-0.0034X_{1}^{2}-0.0038X_{2}^{2}-0.1542X_{3}^{2},\\ DPPH=-364.232+3.1961X_{1}+4.8672X_{2}+45.2018X_{3}-0.0167X_{1}X_{2}+0.0075X_{1}X_{3}-0.0179X_{2}X_{3}-0.0359X_{1}^{2}-0.0307X_{2}^{2}-2.2141X_{3}^{2},\\ FRAP=-420.953+3.8582X_{1}+5.9958X_{2}+42.4509X_{3}-0.0144X_{1}X_{2}+0.0662X_{1}X_{3}-0.0041X_{2}X_{3}-0.0596X_{1}^{2}-0.0404X_{2}^{2}-2.2589X_{3}^{2}. \end{array}$$

#### 3.1.2. Analysis of Regression Coefficients and Response Surface Plots

**Figure 1:**
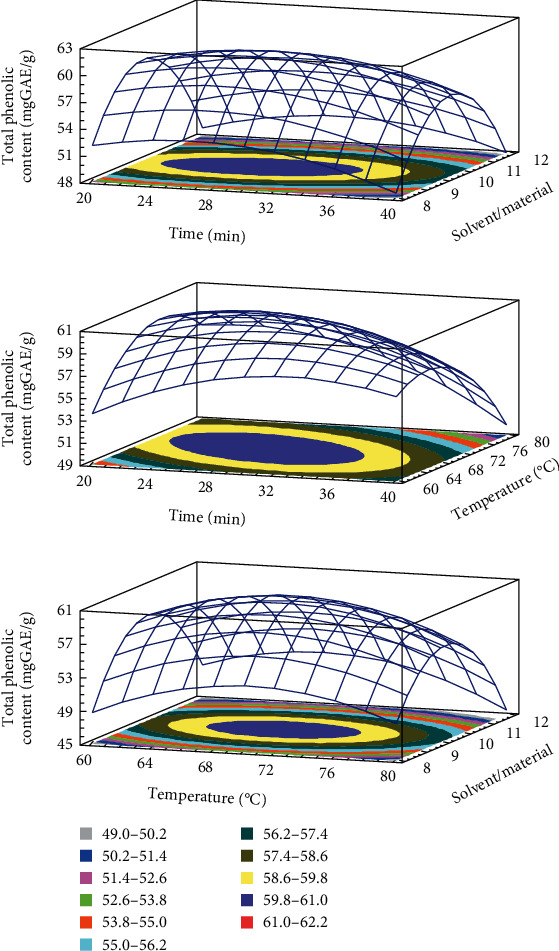
3D response surface plots showing the interactions of various factors affecting the yield of TPC.

**Figure 2:**
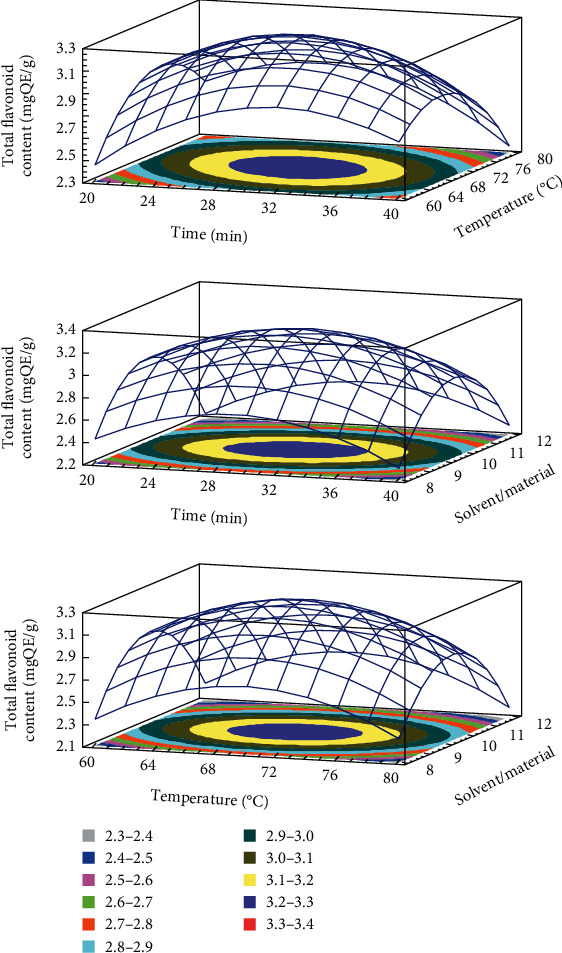
3D response surface plots showing the interactions of various factors affecting the yield of TFC.

**Figure 3:**
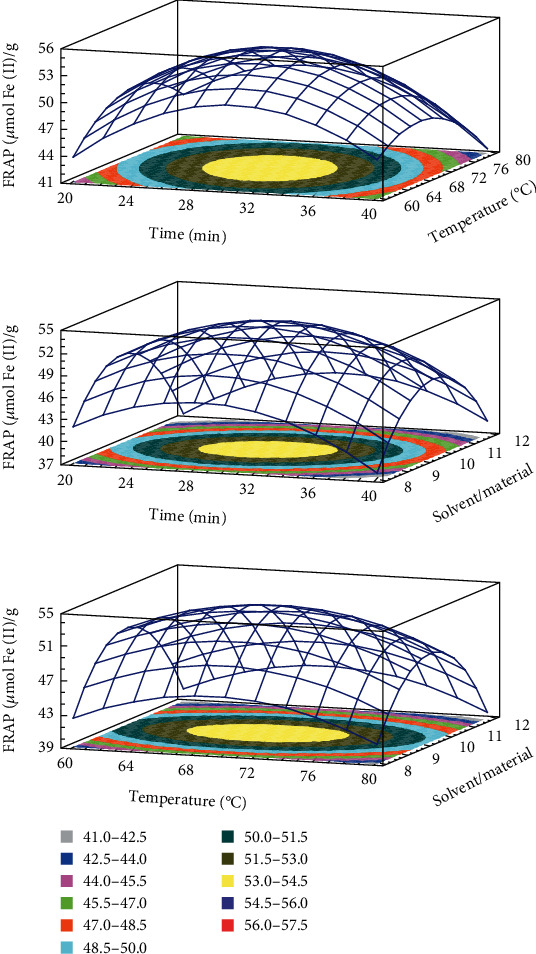
3D response surface plots showing the interactions of various factors affecting the antioxidant activity by FRAP assay.

**Figure 4:**
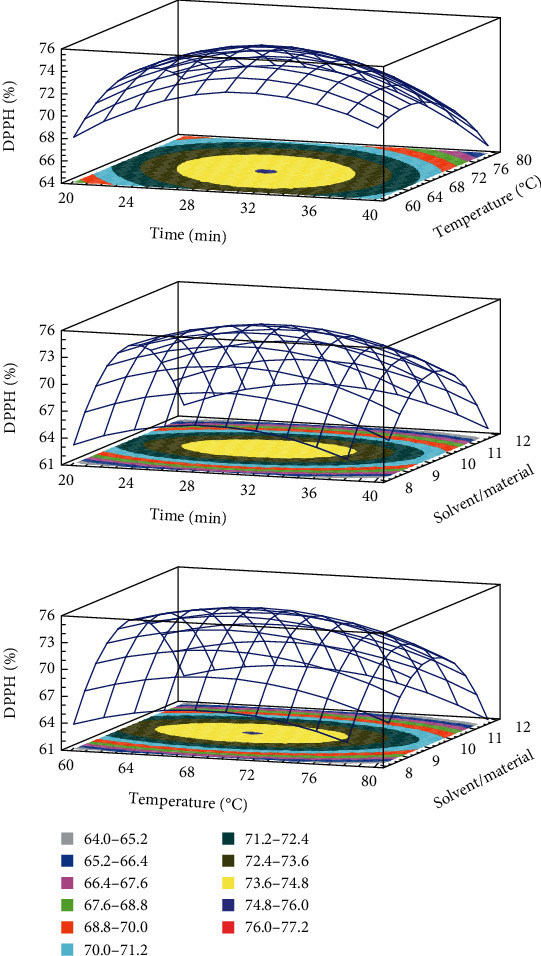
3D response surface plots showing the interactions of various factors affecting the antioxidant activity by inhibition of DPPH.

The developed model for TPC, TFC, DPPH, and FRAP obtained from the multiple linear regressions are reported. The visualization of the statistical significance of the independent variables on the dependent variables was facilitated by the generated surface contour plots (Figures 1–4). To improve the extraction efficiency in this research, the ultrasonication time was controlled from 20 to 40 minutes. At 29.38 minutes of ultrasonication, the overall maximum antioxidant properties were achieved. It may be due to the 20 kHz wave that led to the cavitation bubble impact may help to disrupt the cell wall. Additionally, erosion—a localized form of tissue damage—could be caused by sonicated waves on plants, which is also a result of ultrasound. The collapse of the cavitation bubbles on the outside of plant tissues may also be the reason for this degradation. The extraction yield is increased by allowing it to be easier for the solvent to contact the damaged area [32]. Previously, it was stated that a maximum phenolic yield was obtained from pumpkin at a sonication duration of approximately 27 minutes [33]. The antioxidant compound and pectin yield from white fruit dragon peel also reached a maximum when the extraction time was 30 min [34]. However, extending the sonication period may result in faster movement of molecules, which could change the structure of antioxidant substances and cause their disintegration [35, 36]. As a result, it might not be a good idea to use an extended exposure time with a reduced extraction yield for industrial manufacturing.

The temperature range of 60 to 80°C was used in this investigation. The release of antioxidants is enhanced by the heat effects because there is a greater breakdown of the plant's cell wall [37]. In addition, this is because the vapor pressure of the heated liquid is elevated, which allows cavitation to be achieved at lower ultrasound intensity, thus reducing the strength of shear forces in the vicinity of the bubble [38]. The heat action also contributed to the swelling of plant material and made it softer for releasing bioactive compounds from plant matrix to solvent [18, 39]. The extracted biological substance increased as the temperature rose from 60 to 70°C. However, the antioxidant yield was reduced when the temperature rose over 70°C. It follows that as the temperature rises, the solubility and diffusivity of solids derived from plant material may also increase. Additionally, a higher temperature may destabilize the molecule, which would reduce the effectiveness of the extraction process, especially the heat-sensitive compound [40]. At this condition, it was also found that it could improve the extraction yield of several materials such as butterfly pea flower [41], banana peel powder [25], and red dragon peel [35]. Moreover, it is important to choose effective extraction temperatures that preserve the stability of phenolic compounds because it has been demonstrated that temperatures above 70°C in particular cause fast polyphenol degradation [42]. It is also crucial to note that the types of polyphenol chemicals present in the extract or plant matrix affect how sensitive a sample is to temperature-induced polyphenol degradation.

The ability of the solvent system to penetrate the raw materials is improved by ultrasound waves, which is a key factor in improving extraction efficiency [31, 43, 44]. A larger solvent volume will more effectively dissolve the target chemicals, increasing the extraction yield. The higher the ratio, the higher the total amount of solids obtained, despite the solvent used, according to mass transfer principles [45]. Since the constituents can dissolve more efficiently when there is a concentration difference between the external solvent and the inner plant cell because of the high solvent/material ratio, the mass transfer rate and extraction yield are accelerated [25, 44, 46, 47]. The effectiveness of ultrasonication may be related to the fact that it promotes solute mass transfer to the extraction solvent while enhancing oxygenation and disintegration without significantly degrading the solvent. The solvent/material ratio in the current ultrasound-assisted extraction study is in concordance with an earlier study on quercetin extraction from shallot peel, where a solvent/material ratio was chosen for the optimization process [48].

#### 3.1.3. Validation of the Model

**Figure 5:**
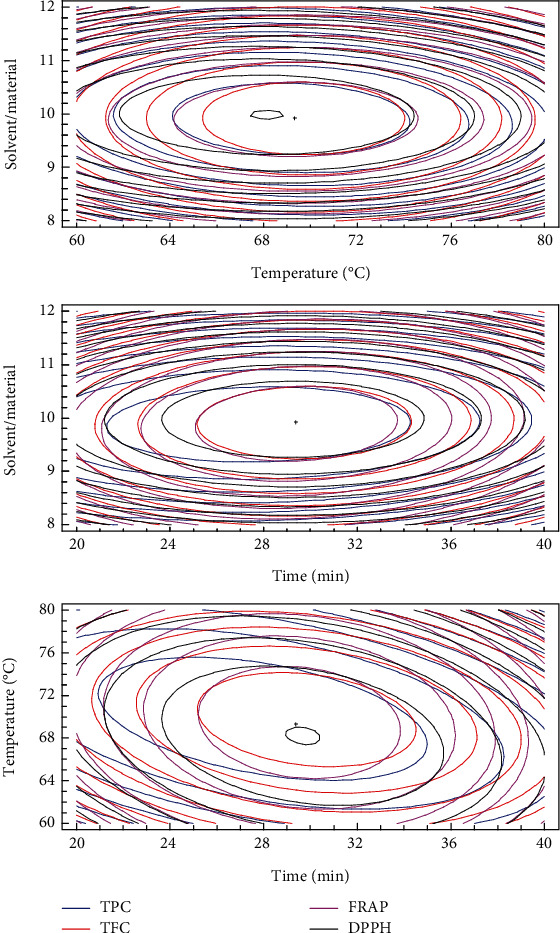
The overlay contour plot of 4 responses (TPC, TFC, DPPH, and FRAP).

The overlay contour plot indicated that a temperature of 69°C and solvent/material ratio of 9.92 mL/mg for 29.38 minutes were the optimal extraction variables at the highest desirability level (0.9405) (Figure 5). Three extractions were carried out under optimal circumstances to verify the model's accuracy. The experimental and projected extraction rates for TPC, TFC, and antioxidant activities (FRAP and DPPH) did not significantly differ with the percentage of difference being lower than 3% (Table 4). As a result, within the range of extraction variables investigated, the model is capable of reliably predicting the behavior of the response variables. Thus, the extraction temperature, time, and solvent/material proportion were fixed at 29.38 min, 69°C, and 9.92 throughout the rest of the study. In addition, under the optimal extraction condition, the yield of extract of UAE (3.4%) was significantly higher than that obtained from the conventional method (1.2%). Besides that, the lower content of phytochemical and antioxidant activities was found in the sample which was extracted without the aid of ultrasound wave. It is in agreement with recent researches that ultrasound could enhance the extraction yield of various plant materials [14, 15, 18–20, 33, 43].

### 3.2. Process of Codigestion of Corn Starch with Purple Rice Bran Extract

**Figure 6:**
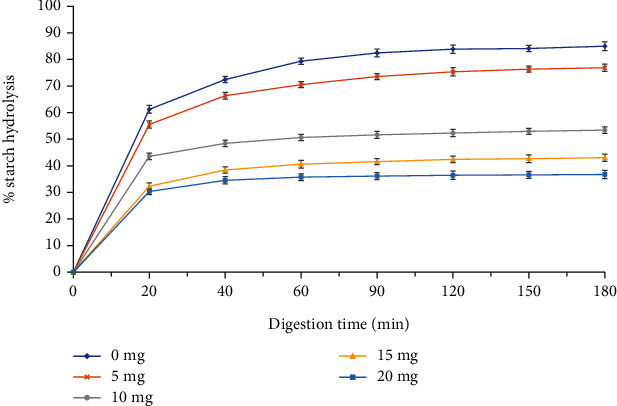
Codigestion cooked corn starch with purple rice bran extract.

The rate of digestion of corn starch supplemented with different levels of purple rice bran extracts is shown in Figure 6. A dose-dependent reduction in the rate of starch digestibility was observed for rice bran extracts that were codigested with cooked corn starch. It could be seen that the digestion rate was initially increased at the former stage; however, as the level of extract increased, a lower percentage of starch hydrolysis was observed. Then, at a later stage, the level of digestion slowed down and slightly increased at the supplementing level of 20 mg extract. While the control sample still increased the rate of hydrolysis and after 180 minutes, almost 80% of cooked corn starch was hydrolysed. Polyphenols could reduce the starch digestibility through various mechanisms, such as complexation together or inhibition of the activity of amylolytic enzyme [21, 49]. In this study, it might be that the biological substance present in the extract reduces the activity of the starch hydrolysis enzyme, leading to different levels of supplementation differing in the percentage of hydrolysis. Dose-dependent was observed, which was similar to the study of Kan et al. [50] about the codigestion process of bread with two kinds of berry extract. They reported that both extracts could reduce the level of hydrolysis of bread, and as the amount of extract increased in codigestion process, the downward trend of starch hydrolysis rate was observed. In addition, the endogenous polyphenols from rice have already been reported to reduce the activity of alpha-amylase enzyme [51]. Therefore, the extracts from “Cẩm” purple rice bran could be potential sources for producing antidiabetic foods.

## 4. Conclusions

Ultrasound-assisted extraction facilitated the augmentation of both antioxidant compounds and antioxidant activities within the “Cẩm” purple rice bran extract. Furthermore, the optimization of extraction conditions was realized, with the most favourable outcomes achieved at an extraction time of 29.38 minutes, a temperature of 69°C, and a solvent-to-material ratio of 9.92. These conditions yielded the highest antioxidant potential for the purple rice bran. Under these optimized parameters, the quantified values for total phenolic content, total flavonoid content, DPPH radical scavenging activity, and FRAP were measured at 60.821 mg GAE/g, 3.2696 mg QE/g, 74.778%, and 54.112 *μ*mol Fe^2+^/g, respectively. Notably, ultrasound-assisted extraction exhibited superior yield compared to conventional methods. Furthermore, the incorporation of the extract into the corn starch digestion process was found to modulate the digestion rate. Specifically, the lowest digestibility was observed when 20 mg of the extract was introduced to 200 mg of cooked corn starch. These observations underscore the prospective applicability of “Cẩm” purple rice bran extract as a potent raw nutraceutical, attributed to its robust antioxidant potency and digestion-inhibitory capabilities. These findings not only hold promise for the utilization of indigenous purple rice bran in Vietnam but also contribute to the foundational knowledge required for the development of low glycemic index products.

## Figures and Tables

**Table 1:** Actual and coded levels of the variables studied in BBD.

Variables	Level used		
	-1	0	1
*X*_1_: extraction time (min)	20	30	40
*X*_2_: extraction temperature	60	70	80
*X*_3_: ratio of solvent to material	8	10	12

**Table 2:** Antioxidant properties of purple rice bran extract under different conditions based on a Box–Behnken design.

Run	*X*_1_ (min)	*X*_2_ (°C)	*X*_3_	TPC (mgGAE/g)	TFC (mgQE/g)	DPPH (%)	FRAP (*μ*molFe(II)/g)
1	30	70	10	60.180 ± 0.593	3.271 ± 0.062	75.041 ± 0.706	54.345 ± 0.001
2	30	70	10	61.625 ± 0.478	3.287 ± 0.051	73.885 ± 0.488	53.305 ± 0.218
3	30	70	10	61.808 ± 0.500	3.298 ± 0.063	74.731 ± 0.552	54.223 ± 0.428
4	30	70	10	60.626 ± 1.222	3.237 ± 0.005	74.614 ± 0.661	54.108 ± 0.300
5	30	70	10	60.507 ± 0.579	3.351 ± 0.004	75.137 ± 0.197	54.453 ± 0.433
6	30	70	10	59.595 ± 1.121	3.172 ± 0.042	74.842 ± 0.643	53.932 ± 0.676
7	20	60	10	53.382 ± 0.344	2.375 ± 0.070	67.439 ± 0.453	43.259 ± 0.534
8	40	60	10	55.914 ± 0.715	2.701 ± 0.066	70.641 ± 0.724	45.521 ± 0.209
9	20	80	10	58.014 ± 0.502	2.727 ± 0.054	68.799 ± 0.668	45.471 ± 0.635
10	40	80	10	50.152 ± 0.137	2.407 ± 0.082	65.308 ± 0.063	41.979 ± 0.450
11	20	70	8	51.766 ± 1.421	2.459 ± 0.008	64.278 ± 0.595	42.488 ± 0.133
12	40	70	8	48.785 ± 1.048	2.315 ± 0.053	61.990 ± 0.555	37.881 ± 0.504
13	20	70	12	48.819 ± 0.526	2.236 ± 0.033	62.236 ± 0.289	37.592 ± 0.326
14	40	70	12	48.371 ± 0.956	2.248 ± 0.167	60.549 ± 0.574	38.285 ± 0.302
15	30	60	8	49.267 ± 1.227	2.358 ± 0.076	63.290 ± 0.651	42.415 ± 0.052
16	30	80	8	49.115 ± 1.248	2.258 ± 0.110	61.389 ± 0.520	40.918 ± 0.501
17	30	60	12	49.267 ± 0.284	2.311 ± 0.025	64.885 ± 0.545	40.887 ± 0.495
18	30	80	12	45.111 ± 0.313	2.168 ± 0.058	61.553 ± 1.000	39.721 ± 0.308

Note: *X*_1_: extraction time (min); *X*_2_: extraction temperature; *X*_3_: ratio of solvent to material.

**Table 3:** Analysis of variance and regression coefficients of the predicted model for response variables.

Model parameters	TPC (mgGAE/g)	TFC (mgQE/g)	DPPH (%)	FRAP (*μ*mol Fe(II)/g)
Intercept	-434.310^∗^	-36.468^∗^	-364.232^∗^	-420.953^∗^
Linear				
*X*_1_	2.9267^∗^	0.2947^∗^	3.1961^∗^	3.8582^∗^
*X*_2_	6.5341^∗^	0.5820^∗^	4.8672^∗^	5.9958^∗^
*X*_3_	45.752^∗^	3.0372^∗^	45.2018^∗^	42.4509^∗^
Interaction				
*X*_1_*X*_2_	-0.0260^∗^	-0.0016^∗^	-0.0167^∗^	-0.0144^∗^
*X*_1_*X*_3_	0.0317^∗^	0.0019	0.0075	0.0662^∗^
*X*_2_*X*_3_	-0.0500^∗^	-0.0005	-0.0179	0.0041^∗^
Quadratic				
*X*_1_^2^	-0.0256^∗^	-0.0034^∗^	-0.0359^∗^	-0.0596^∗^
*X*_2_^2^	-0.0380^∗^	-0.0038^∗^	-0.0307^∗^	-0.0404^∗^
*X*_3_^2^	-2.1830^∗^	-0.1542^∗^	-2.2141^∗^	-2.2589^∗^
*R* ^2^	97.55	97.44	98.19	99.40
Adjusted *R*^2^	97.05	96.92	97.83	99.28
*P* value	0.0000	0.0000	0.0000	0.0000
*F* value	194.79	186.05	250.33	813.94
*P* value of lack-of-fit	0.8703	0.2868	0.0512	0.5054

^∗^ *P* value < 0.05.

**Table 4:** Optimize desirability and model validation.

Optimal extraction variables		Response variables	Predicted value	Experimental value^a^	% difference^b^	Control sample^c^
Time (min)	29.38	TPC (mgGAE/g)	60.82	59.44 ± 0.36	2.33	45.24 ± 0.45
Temperature (°C)	69	TFC (mgQE/g)	3.27	3.25 ± 0.09	0.71	2.12 ± 0.09
Solvent/material	9.92	FRAP [*μ*mol Fe(II)/g]	54.11	55.27 ± 0.96	2.89	37.34 ± 1.34
Desirability	0.94	DPPH (%)	74.78	73.44 ± 0.85	1.82	50.56 ± 0.99

^a^Experimental values were recorded when sample was extracted under selected optimal conditions (three replicates, *n* = 3); ^b^percentage difference between predicted and actual values; ^c^control sample was conducted under optimal conditions without ultrasound treatment (three replicates, *n* = 3).

## Data Availability

Data is contained within the article.
